# Increased Ratio of CD14^++^CD80^+^ Cells/CD14^++^CD163^+^ Cells in the Infrapatellar Fat Pad of End-Stage Arthropathy Patients

**DOI:** 10.3389/fimmu.2021.774177

**Published:** 2021-11-26

**Authors:** Shuhe Ma, Kosaku Murakami, Rintaro Saito, Hiromu Ito, Koichi Murata, Kohei Nishitani, Motomu Hashimoto, Masao Tanaka, Masahi Taniguchi, Koji Kitagori, Shuji Akizuki, Ran Nakashima, Hajime Yoshifuji, Koichiro Ohmura, Akio Morinobu, Tsuneyo Mimori

**Affiliations:** ^1^ Department of Rheumatology and Clinical Immunology, Kyoto University Graduate School of Medicine, Kyoto, Japan; ^2^ Center for Cancer Immunotherapy and Immunobiology, Kyoto University Graduate School of Medicine, Kyoto, Japan; ^3^ Department of Orthopaedic Surgery, Kyoto University Graduate School of Medicine, Kyoto, Japan; ^4^ Department of Orthopaedic Surgery, Kurashiki Central Hospital, Okayama, Japan; ^5^ Department for Advanced Medicine for Rheumatic Disease, Kyoto University Graduate School of Medicine, Kyoto, Japan; ^6^ Department of Clinical Immunology, Osaka City University Graduate School of Medicine, Osaka, Japan; ^7^ Ijinkai Takeda General Hospital, Kyoto, Japan

**Keywords:** osteoarthritis, rheumatoid arthritis, monocytes/macrophage, inflammation, infrapatellar fat pad (Hoffa’s)

## Abstract

**Objectives:**

This study sought to identify the ratio of M1/M2 cells in the infrapatellar fat pads (IFP) and subcutaneous fat tissues (SC) of osteoarthritis (OA) and rheumatoid arthritis (RA) patients. The clinical features of OA and RA patients treated with or without biological disease-modifying anti-rheumatic drugs (bDMARDs) were also assessed.

**Methods:**

IFP and SC were collected from patients with OA and RA who are undergoing total knee arthroplasty (TKA). CD14-positive cells were then isolated from these samples. Flow cytometry was used to determine the number of CD14^++^CD80^+^ cells and CD14^++^CD163^+^ cells. The expression levels of lipid transcription factors, such as sterol regulatory element-binding protein 1 (SREBP1) and liver X receptor alpha (LXRA), and inflammatory cytokines were also evaluated.

**Results:**

Twenty OA patients and 22 RA patients were enrolled in this study. Ten of the RA patients (45.4%) received bDAMRDs before TKA. On average, a fivefold increase in the number of CD14-positive cells and lower expression levels of *SREBP1C* and *LXRA* were observed in OA IFP relative to OA SC; however, these results were not obtained from the RA samples. The median ratio of CD14^++^CD80^+^ cells/CD14^++^CD163^+^ cells of OA IFP was 0.87 (0.76–1.09, interquartile range), which is higher to that of OA SC with a lower ratio (*p* = 0.05835).

**Conclusions:**

The quantity and quality of CD14-positive cells differed between IFP and SC in arthropathy patients. To our knowledge, this is the first study to characterize the ratio of M1/M2 cells in the IFP and SC of end-stage OA and RA patients. The increased ratio of CD14^++^CD80^+^ cells/CD14^++^CD163^+^ cells in the IFP from patients with OA and RA treated with bDMARDs indicated that inflammation was localized in the IFP. As adipose tissue-derived innate immune cells were revealed as one of the targets for regulating inflammation, further analysis of these cells in the IFP may reveal new therapeutic strategies for inflammatory joint diseases.

## Introduction

Osteoarthritis (OA) and rheumatoid arthritis (RA) affects millions of people worldwide, with one in three people over the age of 65 having OA (1), and every five out of a thousand people suffer from RA (2). OA and RA are characterized by joint pain; however, in some cases, they are characterized by deformity owing to inadequate therapeutic strategies. The progression of OA can be seen as multiple reasons, such as mechanical stress to the cartilage (3, 4), subchondral bone remodeling (5), biochemical cascades (6), and inflammation (5, 6). On the other hand, RA is an autoimmune disease that causes inflammation in the synovial tissue (3, 7). Disease-modifying anti-rheumatic drugs (DMARDs), such as biological DMARDs (bDMARDs), are used to treat RA (8); however, currently, no disease-modifying therapeutics is available for OA patients. Thus, elderly patients with OA or RA sometimes opt for total knee arthroplasty (TKA) to restore joint function.

**Figure 1:**
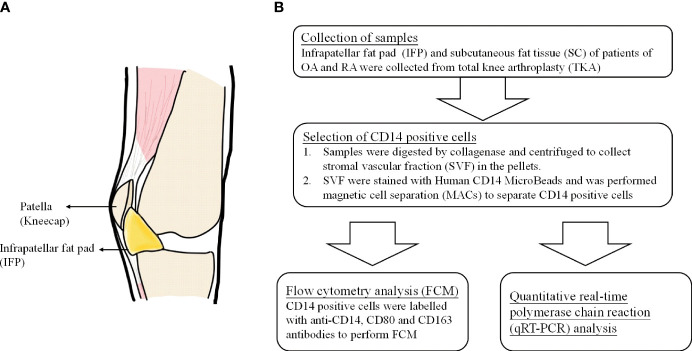
**(A)** The anatomical location of infrapatellar fat pads. **(B)** Flow chart of the experimental method.

Adipose tissue participates in inflammatory processes because it is a source of cytokines, chemokines, and adipokines (9). The infrapatellar fat pad (IFP or Hoffa’s fat pad) is an adipose tissue depot located within the knee joint and is surrounded by synovium, cartilage, and bone (10). The anatomical location of the IFP is displayed in **Figure 1A** (11). As described by Jiang et al., the IFP is sandwiched between the patellar retinacula and the patellar tendon anteriorly and the trochlear surface of the femur posteriorly on the horizontal perspective. In contrast, on the vertical perspective, the IFP is located between the patella, femoral condyle, and intercondylar notch (12). Functionally, the IFP is involved in shock absorption, lubrication, and synovial fluid secretion. Furthermore, it is an important site for inflammation in patients with pain due to OA. Notably, serious inflammation in the IFP is often associated with severe pain in such patients (12, 13). In this study, subcutaneous fat tissue (SC) is defined as fat tissues located under the epidermis and dermis but not found in the IFP tissues located in the lesion parts of the knees.

Adipose tissue comprises the stromal vascular fraction (SVF) and adipocytes, and adipose tissue macrophages and monocytes can be collected from the SVF (10). Adipose tissue macrophages (ATMs) in the IFP demonstrate aspects of both pro- and anti-inflammatory phenotypes *in vitro*, with most cells expressing the M2 marker, CD206, but secreting interleukin-6 (IL-6), tumor necrosis factor-alpha (TNF-α), and relatively low amounts of interleukin-10 (IL-10), similar to M1 cells (13–16). ATMs have been revealed to play important roles in both physiological processes and pathological mechanisms in adipose tissue—for instance, the proportion of ATM in lean mice was estimated to be less than 10% of all adipose tissues; however, in leptin-deficient mice, this proportion increased to over 50% (17). Unlike ATMs that are distributed throughout uninflamed adipose tissue and provide limited inflammatory activity in the lean state (18), ATMs in obese adipose tissue surround and consume dead adipocytes and exhibit a pro-inflammatory phenotype (19, 20). Furthermore, bigger amounts of TNF-α and IL-6 are secreted by ATMs in obese people (9), and a twofold increase in the release of IL-6 was observed in the IFP compared to the SC in patients with OA (21). However, the differentiation patterns of M1 and M2 cells in the IFP of arthropathy patients have not been studied.

To our knowledge, this is the first study to reveal the differentiation pattern of CD14^++^CD80^+^ cells and CD14^++^CD163^+^ cells in the IFP and SC of OA and RA patients. Furthermore, as IFP and SC are adipose tissues in the knee joints (**Figure 1A**) and IFP is considered to have a great potential to participate in inflammatory response (13), qRT-PCR was performed to identify the lipid transcription factors related to inflammation to clarify the signature expression pattern of these genes in the IFP and SC of arthropathy patients.

## Materials and Methods

### Sample Collection From Patients With OA and RA

#### Patient Information

This study was approved by the Kyoto University Graduate School and Faculty of Medicine Ethics Committee (approval number G0502-1). Samples of the IFP and the SC were collected during TKA of patients with OA and RA admitted at Kyoto University Hospital from February 2018 to April 2020.

#### SVF Collection and Human CD14 Magnetic Cell Separation

Briefly, 2 g of fat tissue from each sample was digested in 5 ml of collagenase medium [0.1 mg collagenase IA (#C2674, Sigma Japan) in 1 ml RPMI medium (#52400, Gibco)] at 37°C for at least 1 h with rotation. The digested tissue was mashed through a 70-µm cell strainer with 2 ml syringe plungers and centrifuged at 18°C 620 × *g* for 3 min. The obtained pellets, labeled as the SVF, were washed twice in phosphate-buffered saline (PBS) *via* centrifugation at 18°C 620 × *g* for 3 min. Thereafter, they were rewashed in a magnetic cell separation (MACs) buffer solution of 0.5 M ethylenediaminetetraacetic acid (EDTA) in PBS containing 0.1% bovine serum albumin (BSA) *via* centrifugation at 18°C 300 × g for 10 min and subjected to MACs with human CD14 MicroBeads (#130-050-201, Miltenyi Biotec).

### Flow Cytometry Analysis

#### Staining Strategy

CD14-positive cells were labeled with anti-CD14, anti-CD80, and anti-CD163 antibodies in 0.1% BSA and 0.5 M EDTA in PBS, with isotype and unstained controls. The following conjugated antibodies were used: anti-human CD14 (HCD14, APC, BioLegend), anti-human CD80 (2D10, PE, BioLegend), and anti-human CD163 (RM3/1, PerCP/Cyanine5.5, BioLegend). The following isotype controls were used: mouse IgG1, κ isotype control (FC) antibody (MOPC-21, APC, BioLegend); mouse IgG1, κ isotype control antibody (MOPC-21, PE, BioLegend); and mouse IgG1, κ isotype control antibody (MOPC-21, PerCP/Cyanine5.5, BioLegend). After washing, the cells were analyzed with a LSRFortessor cytometer (BD), and data were analyzed using FlowJo 10.4 software.

#### Gating Strategy

The gating strategy and fluorescence minus one are presented in **Supplementary Figures S1**, **S2**. Representative APC-FSC two-parameter dot plots of gate 1 among unstained, isotype control, and fully stained samples were presented. Cells gathered in neither unstained nor isotype controls in fully stained samples were allotted to gate 2. In gate 2, representative two-parameter dot plots of PE-APC and PerCP/Cyanine5.5-APC of samples stained by isotypes and antibodies were presented. Compared with the isotype control, positive plots were counted as “CD14^+^, CD80^+^” and “CD14^+^, CD163^+^” to calculate the proportions of CD14^++^CD80^+^ cells and CD14^++^CD163^+^ cells in CD14-positive cells.

### Quantitative Real-Time Polymerase Chain Reaction

Total RNA was isolated with Isogen (#315-02504, Nippon Gene) and RNeasy Mini Kit (#74104, Qiagen). After DNAse treatment (#M6101, Promega), cDNA was prepared using an iScript cDNA Synthesis Kit (#1708890, BIO-RAD) according to the protocol of the manufacturer. qPCR was performed using TB Green Premix Ex Taq GC (#RR071A, TaKaRa) in a 7500 Real Time PCR System (Applied Biosystems). The following qPCR conditions were employed: one cycle for the initial denaturation stage at 95°C for 30 s, 70 cycles for the PCR stage with denaturation at 95°C for 10 s, and annealing at 60°C for 34 s; the melt curve stage was set according to the instructions for Takara TB Green for the 7500 Real-Time PCR System. The cell counts of the operation samples used in RNA extraction and qPCR are displayed in **Supplementary Table S1**. The sequences of the primers are listed in **Supplementary Table S2** (22–25).

### Statistical Analyses

Patient information is presented as median values with interquartile ranges (IQR). For qPCR, the 2^−ΔΔCT^ method was used to quantify the data. As the collected data did not follow normal distribution, Mann–Whitney *U*-test was utilized for statistics to describe differences between the groups. Furthermore, as sample sizes were the same within the same patients or the same SVF, paired-sample Wilcoxon signed-rank test was utilized to perform a more rigorous analysis. The statistical data were calculated using Origin 9.0 software (Originlab).

## Results

### The IFP of Arthropathy Patients Has a Higher Proportion of CD14+ Cells Than the SC

The patients included in this study were arthropathy patients who underwent TKA. These patients were divided into OA, RA treated without bDMARDs, and RA treated with bDMARDs groups, as shown in **Table 1**. Twenty OA patients (group A) [15 females; median (IQR) age, 77.4 (74.7–81.3) years], 12 RA patients who were not treated with bDMARDs (group B) [9 females; 67.6 (66.5–70.5) years], and 10 RA patients who were treated with bDMARDs (group C) [10 females; 68.2 (46.5–76.0) years] were included in the study. Information regarding C-reactive protein (CRP), erythrocyte sedimentation rate, total cholesterol, high-density lipoprotein, rheumatoid factor, anti-citrullinated protein antibodies, tender joint count, swollen joint count, patient’s and physician’s global assessment visual analog scale, and simplified disease activity index and the use of methotrexate (MTX) and prednisolone were described. OA patients who opted for surgery tended to be older, with a significance found between OA and both RA groups. The median body weight (kg) of patients who underwent surgery in these groups was 63.5, 51.8, and 59.8 kg, respectively, with a significance found between the group of RA patients who were not treated with bDMARDs and the other two groups. To gain more insights on whether gender is a factor in this significance, female and male patients were divided within each group. As shown in **Table 1**, female body weight had the same significance as that found for total patients, revealing a bias in body weight between genders. As the body mass index (BMI) of all members of the “RA without bDMARDs treatment” group was below 25 kg/m^2^, the *p*-values of the overweight population between this group and the other two groups could not be calculated.

**Table 1:** Patient characteristics.

Patient groups	Group A	Group B	Group C		*p*-values between groups						
	OA	RA without bDMARDs	RA with bDMARDs		A and B		B and C			A and C	
Total patients (*n*)	20	12	10	Age (years)		**0.0033**		0.6628			**0.0040**
Female patients (*%*)	75%	75%	100%	Height (cm)		0.3658		0.1855			0.9741
Age (years)	77.4 (74.7–81.3)	67.6 (66.5–70.5)	68.2 (46.5–76.0)	Body weight/total (kg)		**0.0023**		**0.0358**			0.5661
Height (cm)	153.3 (149.7–160.7)	158.3 (154.3–162.0)	153.8 (152.0–156.7)	Body weight/F (kg)		**0.0087**		**0.0101**			0.8244
Body weight/total (kg)	63.5 (55.4–70.3)	51.8 (47.8–55.4)	59.8 (56.6–63.7)	Body weight/M (kg)		0.0736	Not assessed			Not assessed	
Body weight/F (kg)	60.9 (52.8–68.1)	48.8 (45.4–53.3)	59.8 (56.6–63.7)	BMI >24.99	Not assessed		Not assessed				0.3929
Body weight/M (kg)	70.4 (70.2–75.8)	60.1 (55.1–63.6)	Not applicable	CRP (mg/dl)		0.1717		0.5942			0.4574
BMI (kg/m^2^)	26.1 (24.3–28.0)	19.9 (19.5–23.6)		ESR (mm/h)		0.1327		0.5278			0.4539
% of Obesity	65.0%	8.3%	70.0%	TC (mg/dl)		0.7958		0.1273			0.0926
CRP (mg/dl)	0.0 (0.0–0.13)	0.1 (0.0–1.6)	0.05 (0.0–0.4)	HDL (mg/dl)		0.9892		0.3053			0.1498
ESR (mm/h)	23.0 (10.8–30.3)	27.0 (18.5–50.5)	26.5 (6.0–44.0)	Disease duration (years)				0.2702			
TC (mg/dl)	193.0 (185.8–224.0)	197.0 (173.8–225.5)	221.5 (209.0–234.0)	% of RF positive				0.2530			
HDL (mg/dl)	63.0 (57.3–73.8)	62.0 (51.0–76.0)	77.0 (64.0–83.0)	% of ACPA positive				0.1273			
Disease duration (years)		11.7 (5.6–19.2)	15.1 (12.0–22.5)	% MTX use				0.4003			
% of RF positive		78.6	100	MTX dose (mg/week)				0.4205			
% of ACPA positive		71.4	100	% PSL use				1.0000			
% MTX use		42.9	66.7	PSL dose (mg/day)				0.7277			
MTX dose (mg/week)		0 (0–8.5)	6.0 (0–11.0)	TJC				0.0659			
% PSL use		28.6	33.3	SJC				0.8313			
PSL dose (mg/day)		0 (0–2.5)	0 (0–4.5)	PtGA VAS (cm)				0.8847			
TJC		2.0 (1.0–2.0)	1.0 (0–2.0)	PhGA VAS (cm)				0.2461			
SJC		1.0 (1.0–2.0)	1.0 (1.0–2.0)	CRP (mg/dl)				0.0755			
PtGA VAS (cm)		3.6 (2.1–6.9)	4.0 (1.9–6.7)	SDAI				0.2043			
PhGA VAS (cm)		3.3 (1.9–3.9)	2.0 (1.2–3.6)								
SDAI		11.5 (8.0–15.7)	10.7 (6.2–11.3)								

Data are presented as medians and interquartile ranges. Statistics between groups were assessed using Mann–Whitney U-tests or Fisher’s exact tests. Bold values indicates significance.

OA, osteoarthritis; RA, rheumatoid arthritis; bDMARDs, biological disease-modifying anti-rheumatic drugs (tocilizumab, n = 4; etanercept, n = 3; golimumab, n = 2; sarilumab, n = 1); F, female; M, male; BMI, body mass index; CRP, C-reactive protein; ESR, erythrocyte sedimentation rate; TC, total cholesterol; HDL, high-density lipoprotein; RF, rheumatoid factor; ACPA, anti-citrullinated protein antibodies; MTX, methotrexate; PSL, prednisolone; TJC, tender joint count; SJC, swollen joint count; PtGA, patient’s global assessment; VAS, visual analog scale; PhGA, physician’s global assessment; SDAI, simplified disease activity index.

**Figure 2:**
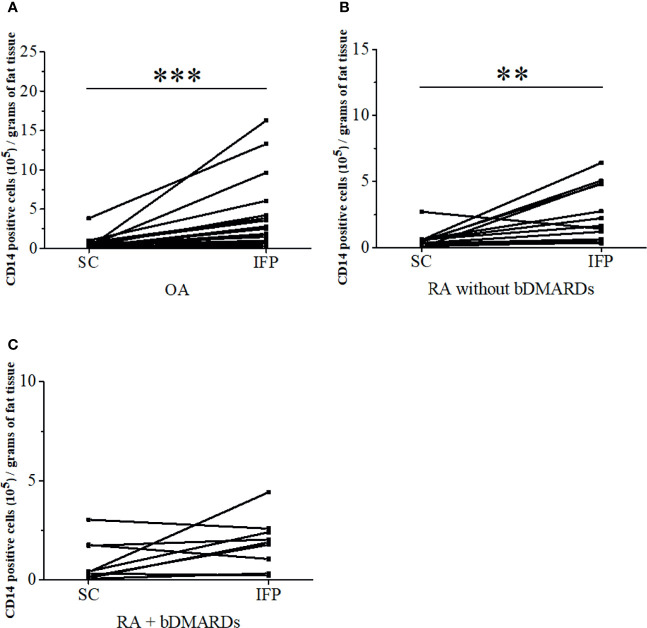
The infrapatellar fat pads (IFP) of arthropathy patients has a higher proportion of CD14-positive cells than the subcutaneous fat tissues. CD14-positive cells per gram of SC or IFP in the osteoarthritis (OA) **(A)**, rheumatoid arthritis (RA) without bDMARDs **(B)**, and RA with bDMARDs **(C)**. Samples were obtained during total knee arthroplasty, digested in collagenase medium to obtain the stromal vascular fraction, and stained with human CD14 antibody to separate adipose-tissue-resident CD14-positive cells. **p < 0.01 and ***p < 0.001, as determined by the paired-sample Wilcoxon signed-rank test to identify differences between SC and IFP OA. n = 20; RA without bDMARDs, *n* = 12; RA with bDMARDs, *n* = 10.

CD14-positive cells from IFP and SC were assessed. The strategies to obtain CD14-positive cells and a schematic for flow cytometry and qPCR are displayed in **Figure 1B**. The amount of CD14-positive cells per gram of IFP was compared to that of SC for the same patients in each group (**Figure 2**). In the OA group, the amount of CD14-positive cells (×10^5^) in the IFP of median (IQR) was 2.57 (0.96–4.13); however, this amount decreased to 0.28 (0.067–0.55) in the SC, with a *p*-value less than 0.0001 depicting significance. In RA patients who were not treated with bDMARDs, the number of CD14-positive cells (×10^5^) in the IFP was 1.64 (0.61–4.84); however, this number decreased to 0.42 (0.20–0.58) in the SC, with significance found (*p* = 0.00792). In RA patients treated with bDMARDs, the number of CD14-positive cells (×10^5^) in the IFP was 1.89 (0.69–2.49); however, this number decreased to 0.39 (0.13–1.74) in the SC. Statistics on the mean (standard deviation, SD) of CD14-positive cells per gram of fat tissue in each group are displayed in **Supplementary Figure S3A**.

### CD14^++^CD80^+^ and CD14^++^CD163^+^ Cell Ratio Increased by 1.36-Fold in OA IFP

**Figure 3:**
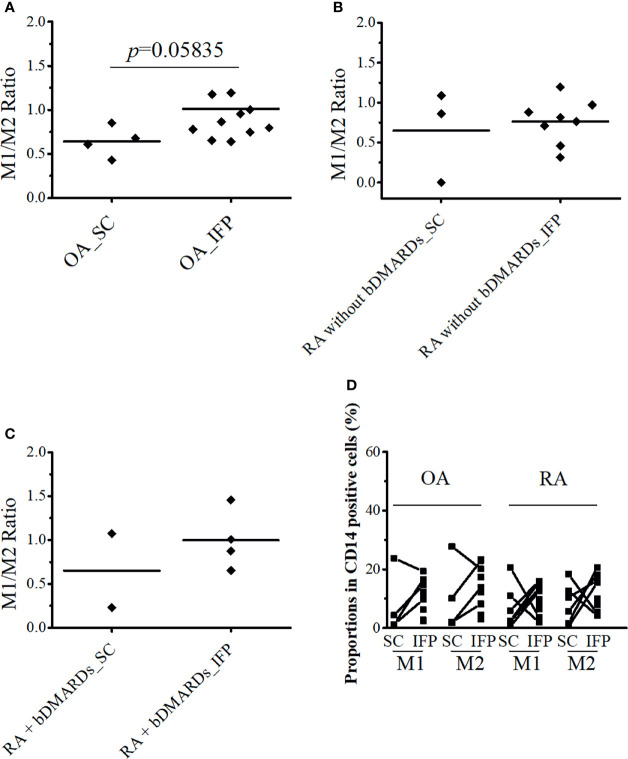
CD14^++^CD80^+^ and CD14^++^CD163^+^ cell ratio increases by 1.36-fold in osteoarthritis (OA) infrapatellar fat pads (IFP). **(A–C)** Ratio of CD14^++^CD80^+^/CD14^++^CD163^+^ cells in the infrapatellar fat pads (IFP) and subcutaneous fat tissues (SC) from the OA, rheumatoid arthritis (RA) without bDMARDs, and RA treated with bDMARDs groups. To calculate ratios and proportions, adipose-resident CD14-positive cells were stained with anti-human CD80, CD163, and CD14 antibodies and evaluated using flow cytometry. **(D)** Proportions of CD14^++^CD80^+^ and CD14^++^CD163^+^ cells in the SC and IFP tissues of OA and RA patients (SC OA, *n* = 4; SC RA, *n* = 6; IFP OA, *n* = 11; IFP RA, *n* = 13). The x-axis displays samples from IFP and SC grouped by OA and RA, and the y-axis displays the proportions of CD14^++^CD80^+^ cells or CD14^++^CD163^+^ cells in CD14-positive cells. Lines connecting the dots between SC and IFP indicate samples from the same patients. Gating strategies for obtaining CD14^++^CD80^+^ cells and CD14^++^CD163^+^ cells as well as fluorescence minus one are presented in **Supplementary Figures S1** and **S2**. Statistics were calculated by the paired-sample Wilcoxon signed-rank test between CD14^++^CD80^+^ and CD14^++^CD163^+^ cells from the same patients. The Mann–Whitney *U*-test was used for analysis between different groups.

CD14-positive cells from the IFP and SC of all three groups were assessed. CD14^++^CD80^+^ cells and CD14^++^CD163^+^ cells were considered as M1 and M2 macrophages. As described in **Figure 3A**, the ratio [median (IQR)] of CD14^++^CD80^+^ cells/CD14^++^CD163^+^ cells of OA IFP was 0.87 (0.76–1.09); this ratio was higher to that of OA SC with a lower degree (*p* = 0.05835). In the group of RA patients who were not treated with bDMARDs, the ratio of CD14^++^CD80^+^/CD14^++^CD163^+^ from both the IFP and SC was as high as 0.79 (0.65–0.90) and 0.86 (0.43–0.98) (**Figure 3B**). The RA bDMARDs group also had an increased ratio from 0.65 (0.44–0.86) in the SC to 0.94 (0.82–1.12) in the IFP (**Figure 3C**). **Figure 3D** shows that both diseases even had differentiation of CD14^++^CD80^+^ cells and CD14^++^CD163^+^ cells in the IFP. Statistics on the mean (SD) of M1/M2 ratio as well as their proportions in CD14-positive cells in each group are shown in **Supplementary Figures S3B, C**. The cell numbers in each group are presented in **Table 2**. However, the M1/M2 ratio was 0.59 (0.31–1.11) and was found to be biased to the M2 phenotype in peripheral blood mononuclear cell from RA patients (26).

**Table 2:** CD14^++^CD80^+^ cells and CD14^++^CD163^+^ cells from the IFP and SC in each disease group.

	IFP		SC	
	CD14^++^CD80^+^	CD14^++^CD163^+^	CD14^++^CD80^+^	CD14^++^CD163^+^
OA	9.3 × 10^4^ (7.7 × 10^4^)	1.1 × 10^5^ (1.1 × 10^5^)	3.9 × 10^3^ (4.7 × 10^3^)	5.4 × 10^3^ (5.5 × 10^3^)
	*N* = 11		*N* = 4	
RA without bDMARDs	3.2 × 10^4^ (2.8 × 10^4^)	4.4 × 10^4^ (3.3 × 10^4^)	4.9 × 10^3^ (4.7 × 10^3^)	4.8 × 10^3^ (4.6 × 10^3^)
	*N* = 8		*N* = 3	
RA with bDMARDs	5.7 × 10^4^ (4.0 × 10^4^)	7.4 × 10^4^ (6.3 × 10^4^)	672 (76)	1,646 (948)
	*N* = 4		*N* = 2	

Cell numbers were determined using flow cytometry and are displayed as mean (SD).

IFP, infrapatellar fat pad; SC, subcutaneous fat tissue.

### The Expression of Lipid Transcription Factors Related to Inflammation Significantly Decreases in the IFP

**Figure 4:**
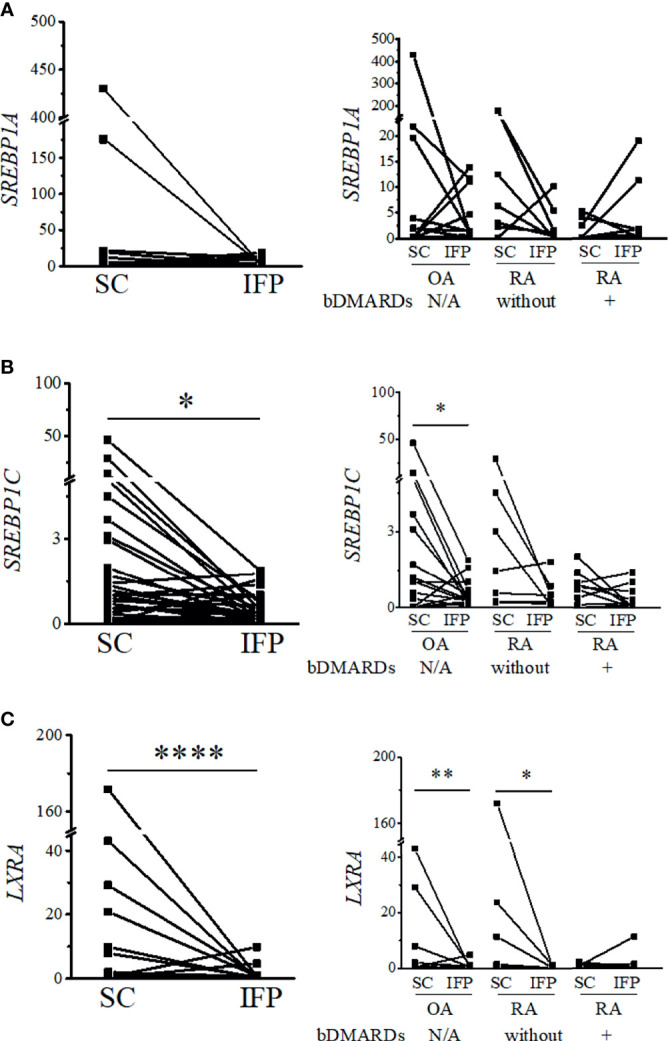
Expression levels of lipid transcription factors related to inflammation decrease in the infrapatellar fat pads (IFP). Expression levels of SREBP1A **(A)**, SREBP1C **(B)**, and LXRA **(C)** in the IFP and subcutaneous fat tissues (SC) from osteoarthritis (OA) and rheumatoid arthritis (RA) patients. The panels to the left contain results comparing the expression levels between SC and IFP without division by diseases. The panels to the right contain results comparing the expression levels of SC and IFP in patients with OA, RA treated with bDMARDs, and RA without bDMARDs treatment. Lines connecting the dots from SC to IFP indicate samples from the same patients. The samples were labeled and separated into CD14-positive cells from the stromal vascular fraction of the IFP and SC from each patient. The median (interquartile range, IQR) expression levels of *SREBP1A*, *SREBP1C*, and *LXRA* in the SC from all patients were 0.31 (0–5.25), 0.98 (0.22–2.98), and 0.69 (0.34–2.12); these expression levels changed to 1.1 (0.09–4.81), 0.30 (0.16–0.71), and 0.039 (0.0085–0.50) in the IFP. The median (IQR) expression levels of *SREBP1C* and *LXRA* in the SC from OA patients were 1.03 (0.03–3.69) and 0.69 (0.34–2.14); these expression levels changed to 0.31 (0.23–0.71) and 0.039 (0.018–0.50) in the IFP. The median (IQR) expression levels of *LXRA* in the SC and IFP from RA patients who were not treated with bDMARDs were 1.04 (0.065–20.89) and 0.017 (0.0067–0.81). Significant differences are displayed in each figure. OA, *n* = 15; RA without bDMARDs, *n* = 7; RA with bDMARDs, *n* = 9, respectively. The 2^−ΔΔCT^ method was used to quantify the qPCR data. *GAPDH* was used as the reference gene. The average *GAPDH* Ct values from all SC samples were used to normalize the Ct values. The Y-axes indicate the relative expression levels of mRNA, which were normalized by *GAPDH*. *p < 0.05, **p < 0.01, and ****p < 0.0001, as determined using the Mann–Whitney *U*-test for differences between the IFP and SC groups and the paired-sample Wilcoxon signed-rank test for differences between IFP and SC within the OA, RA without bDMARDs, and RA with bDMARDs groups.

**Figure 5:**
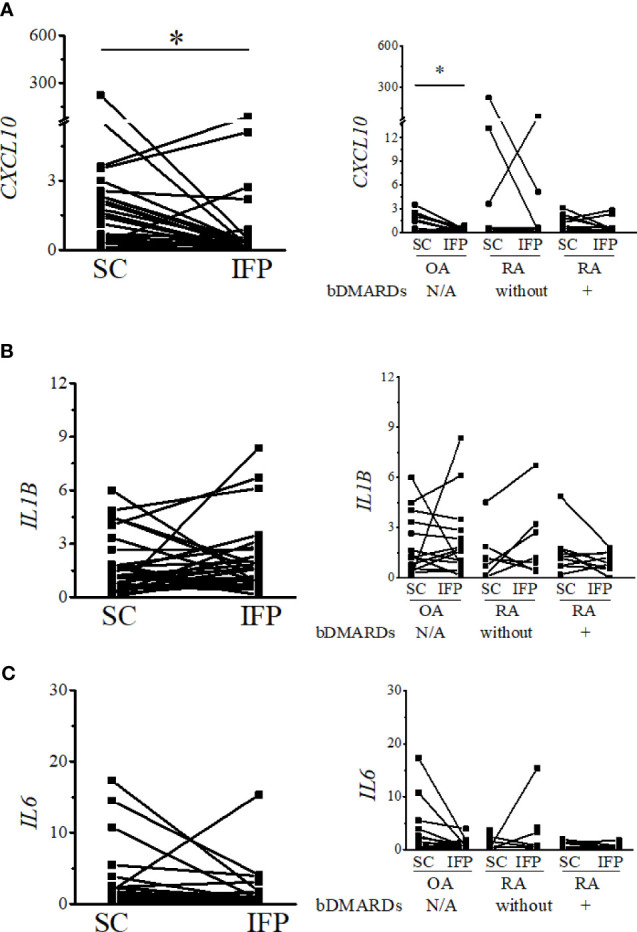
Expression levels of inflammatory cytokines decrease in the IFP. Expression levels of CXCL10 **(A)**, IL1B **(B)**, and IL6 **(C)** in the infrapatellar fat pads (IFP) and subcutaneous fat tissues (SC) from osteoarthritis (OA) and rheumatoid arthritis (RA) patients. The panels to the left contain results comparing the expression levels between SC and IFP without division by diseases. The panels to the right contain results comparing the expression levels of SC and IFP in patients with OA, RA treated with bDMARDs, and RA who were not treated by bDMARDs. Lines connecting the dots from SC to IFP indicate samples from the same patients. The samples were labeled and separated into CD14-positive cells from the stromal vascular fraction of the IFP and SC of each patient. The median (interquartile range) expression levels of *CXCL10* in the SC and IFP from patients were 1.13 (0.23–2.33) and 0.33 (0.10–0.55); the expression level in the SC was 0.98 (0.07–2.33) and changed to 0.17 (0.05–0.42) in the IFP of OA patients. Significant differences are displayed in each figure. OA, *n* = 15; RA without bDMARDs, *n* = 7; RA with bDMARDs, *n* = 9, respectively. The 2^−ΔΔCT^ method was used to quantify the qPCR data. *GAPDH* was used as the reference gene. The average *GAPDH* Ct values from all SC samples were used to normalize the Ct values. The Y-axes indicate the relative expression levels of mRNA, which were normalized by *GAPDH*. *p < 0.05, as determined using the Mann–Whitney *U*-tests for differences between the IFP and SC groups and the paired-sample Wilcoxon signed-rank test for differences between the IFP and SC within the OA, RA without bDMARDs, and RA with bDMARDs groups. Data for the sample whose *CXCL10* 2^−ΔΔCT^ value was 69.2-fold higher than the average were removed from the plot.

As IFP and SC are fat tissues, and IFP is found in an environment related to inflammation, lipid transcription factors related to inflammation, such as SREBPs and LXRs, were of interest in this study. The expression levels of these genes and the inflammatory cytokines produced by CD14-positive cells from the IFP and SC were quantified by qRT-PCR (**Figures 4**, **5**). Statistics on mean (SD) of the expression levels of these genes in CD14-positive cells in each group are presented in **Supplementary Figure S4**. The samples analyzed by qRT-PCR were magnetically sorted CD14-positive cells in the SVF from each SC and IFP sample. Lines connecting the dots from the SC to IFP indicated samples from the same patients. Consequently, as described in the left panel of **Figure 4A**, the relative expression level [median (IQR)] of *SREBP1A* in the CD14-positive cells of SC in all patients was 0.31 (0–5.25); however, in the IFP, this value changed to 1.1 (0.09–4.81), without any significance. As displayed in the left panels of **Figures 4B, C**, **5A**, the respective expression levels of *SREBP1C*, *LXRA*, and *CXCL10* in the CD14-positive cells of SC from whole patients were 0.98 (0.22–2.98), 0.69 (0.34–2.12), and 1.13 (0.23–2.33); however, these levels respectively decreased to 0.30 (0.16–0.71), 0.039 (0.0085–0.50), and 0.33 (0.10–0.55) in the IFP, with *p*-values less than 0.05 depicting significance. These differences were also apparent in patients with OA, with 1.03 (0.03–3.69), 0.69 (0.34–2.14), and 0.98 (0.07–2.33) in the SC, which decreased to 0.31 (0.23–0.71), 0.039 (0.018–0.50), and 0.17 (0.05–0.42) in the IFP, with *p*-values less than 0.05 depicting significance, as shown in the right panels of **Figures 4B, C**, **5A**. In addition, the expression levels of *LXRA* in the RA without bDMARDs treatment group were 1.04 (0.065–20.89) and 0.017 (0.0067–0.81) in the SC and IFP, respectively, which indicated significance. No significant relationship was found between *IL1B* and *IL6* within or among each group (**Figures 5B, C** **)**.

### Inflammation Status Is Influenced by BMI and Age in Arthropathy Patients

The impact of BMI and age on CD14-positive cells, the ratio of CD14^++^CD80^+^ cells/CD14^++^CD163^+^ cells, and the gene expression levels in each disease are highlighted in **Table 3**. Some of the results were not covered in the analysis among whole samples—for instance, in OA patients whose BMI was >25, *IL6* expression was significantly lower in the IFP than the SC; however, in OA patients whose BMI was <25 and in the whole group, this significance disappeared. Furthermore, some results in the whole group showed bias to a certain group in BMI—for example, in the RA without bDMARDs treatment group with BMI >25, *CXCL10* expression was significantly lower in the IFP than SC, while in the RA with bDMARDs treatment group with BMI <25, *LXRA* expression was significantly lower in the IFP than SC. Moreover, patients with BMI >25 in both the OA and RA with bDMARDs treatment groups tended to have higher proportions of CD14-positive cells in the fat tissue. To determine whether age impacts these results, the patients were divided into age groups below or above 75 (**Table 3**), which is indicated as senior people, or were divided into age groups below or above the median age (**Supplementary Table S3**). In **Table 3**, significant differences of CD14-positive cells per gram of fat tissues were found in OA patients who were over 75 years old as well as in the group of RA patients who were not treated by bDMARDs and under 75 years old. On the other hand, the expression levels of *LXRA* and *SREBP1C* had significant differences between SC and IFP in patients under 75 years old of both OA and RA who were not treated by bDMARDs groups. However, in the age groups divided into below or above median age in each disease, the significance of *LXRA* in RA patients who were not treated by bDMARDs was missing (**Supplementary Table S3**), which illustrated the accuracy of age classification in **Table 3**. Overall, inflammation was found to be influenced by BMI and age in arthropathy patients.

**Table 3:** Impact of body mass index (BMI) and age category of osteoarthritis (OA) and rheumatoid arthritis (RA) patients on the related gene expression.

		CD14*-*positive cells (105)/grams of fat tissue	M1/M2 ratio	*SREBP1A*	*SREBP1C*	*LXRA*	*CXCL10*	*IL1B*	*IL6*
BMI <25	OA	**0.0156**	Not assessed	0.6250	0.7500	0.5000	1.0000	0.2500	0.8750
		*N* = 7	*N* = 1	*N* = 4					
	RA without bDMARDs	**0.0068**	1.0000	0.1563	0.15623	**0.0313**	1.0000	0.5625	0.6875
		*N* = 12	*N* = 3	*N* = 6					
	RA with bDMARDs	0.2500	Not assessed	Not assessed					
		*N* = 3	*N* = 0	*N* = 2					
BMI ≥25	OA	**4.88E-04**	0.0952	0.6953	0.1094	0.0977	0.0098	0.2402	**0.0420**
		*N* = 13	*N* = 3	*N* = 11					
	RA without bDMARDs	Not assessed	Not assessed	Not assessed					
		*N* = 1	*N* = 0	*N* = 1					
	RA with bDMARDs	**0.0156**	1.0000	0.4375	0.5625	0.4375	**0.0313**	0.8438	0.3125
		*N* = 7	*N* = 2	*N* = 6					
Age <75	OA	0.0625	0.1143	0.6875	**0.0313**	**0.0313**	0.0938	0.5625	0.5625
		*N* = 6	*N* = 4	*N* = 6					
	RA without bDMARDs	**0.0195**	0.8333	0.2188	0.2188	**0.0313**	0.5625	0.5625	0.8438
		*N* = 11	*N* = 3	*N* = 6					
	RA with bDMARDs	0.4375	Not assessed	1.0000	0.3125	0.0625	0.3125	0.6250	0.1875
		*N* = 6	*N* = 1	*N* = 5					
Age ≥75	OA	**1.22E-04**	Not assessed	0.5703	0.4961	0.0742	0.1953	0.7344	0.0742
		*N* = 14	*N* = 0	*N* = 9					
	RA without bDMARDs	0.33333	Not assessed	Not assessed					
		*N* = 2	*N* = 0	*N* = 1					
	RA with bDMARDs	0.1250	Not assessed	0.5000	0.7500	1.0000	0.2500	0.7500	1.0000
		*N* = 4	*N* = 1	*N* = 3					

The impact of BMI and age in the OA and RA without bDMARDs and RA with bDMARDs groups. The patients were classified into groups with BMI below or over 25 or into age groups below or over 75 years old. The statistics of CD14-positive cells and the gene expression levels in each group were determined by paired-sample Wilcoxon signed-rank test, while the statistics for the M1/M2 ratio in each group were determined using Mann–Whitney U-tests. Data are expressed as p-values between subcutaneous fat tissue and infrapatellar fat pad. Data in bold font indicate significance.

## Discussion

In the present study, samples obtained from patients with OA and RA who underwent knee surgery revealed that the number of CD14-positive cells per gram of IFP was significantly higher than that of the SC. The ratios of CD14^++^CD80^+^ cells/CD14^++^CD163^+^ cells increased between IFP and SC within the OA group and the RA treated with bDMARDs group (*p* = 0.05835 and 0.8170, respectively). In addition, the expression levels of lipid transcription factors related to inflammation were significantly lower in OA IFP than OA SC.

The IFP is demonstrated as an active inflammatory site in arthritis (27). Although the IFP is an adipose tissue, its characteristics differ from other “classic” adipose tissues, such as SC (21). Thus, we compared the features of IFP to those of SC to assess the varying differentiation pattern of macrophages in the IFP in the inflammatory environment. In our study, an average increase of fivefold was found for the number of CD14-positive cells in the IFP relative to that in the SC (**Figure 2**). Therefore, SC and IFP were categorized as “inflammation-low tissue” and “inflammation-high tissue”, respectively. Furthermore, the ratio of CD14^++^CD80^+^ cells/CD14^++^CD163^+^ cells and the expression levels of the genes related to lipid regulation and inflammation were compared to investigate the role of adipose-tissue-resident innate immune cells in the pathological process of arthropathy diseases. However, more samples are needed for this analysis. Furthermore, in addition to staining cells with anti-CD14, CD80, and CD163 antibodies, the quantification of CD68, inducible nitric oxide synthase, and arginase-1 in the adipose-tissue-resident CD14-positive cells by qRT-PCR might provide more reliable results.

As different groups of arthropathy patients have varying characteristics, a comparison between the groups was not performed. The number of CD14-positive cells and ratios of CD14^++^CD80^+^ cells/CD14^++^CD163^+^ cells were increased in the IFP in both the OA group and the RA treated with bDMARDs group. The CRP results based on blood tests (in **Table 1**) revealed a lower inflammation level in the OA [0.0 (0.0–0.13)] and RA treated with bDMARDs [0.05 (0.0–0.4)] groups than the RA without bDMARDs treatment [0.2 (0.0–1.7)] group. Such findings indicate that both OA and RA treated with bDMARDs patients had a lower inflammation level in their blood. However, a higher ratio of CD14^++^CD80^+^ cells/CD14^++^CD163^+^ cells appeared in the IFP of these patients, implying inflammation localization in the IFP. The expression levels of *LXRA* and *SREBP1* displayed different patterns between the IFP and SC in OA and RA patients, and the effects of concurrent or historical administration of MTX could serve as one of the reasons; this is because MTX was revealed to inhibit the amounts of inflammatory signals *via* the JAK1-STAT3 and JAK2-STAT5 transcriptional pathways in human macrophage cell lines (28, 29). In the right panel of **Figure 5A**, the expression levels of *CXCL10* in CD14-positive cells significantly decreased from the SC to IFP in OA; however, in both RA groups, this significance disappeared. As *CXCL10* responds to TNF-α, IFN-α/β/γ, IL-1β, or LPS (30), anti-TNF inhibitors, such as etanercept and golimumab used as bDMARDs (31), can alter *CXCL10* expression. The lower expression of *LXRA* in the IFP can be considered as an inhibitory effect of cytokines (32). The ratios of CD14^++^CD80^+^ cells/CD14^++^CD163^+^ cells in the IFP were relatively higher than those in the SC, which indicated that the IFP was in an inflammatory state. The *p*-value was displayed to show importance, but as the tendency is strong, if more samples can be obtained in the future, the interpretive power is supposed to increase.

To understand the mechanism of LXR downregulation in the IFP, besides inflammatory response genes, the expression levels of genes encoding enzymes involved in unsaturated fatty acid biosynthesis (*e*.*g*., *SCD2*, *FADS1*, *ACOX3*, and *ELOVL5*) and the concentrations of omega-3 fatty acids (*e*.*g*., docosahexaenoic acid and eicosatetraenoic acid) and omega-7 fatty acids (*e*.*g*., 9Z-palmitoleic acid) need to be evaluated (33). Based on the theory that lipid transcription factors participate in the inflammation process, these results would be the first step to see the panorama of “localized lipid immunization.”

This study revealed that, in the IFP of arthropathy patients, abundant CD14-positive cell stores and the ratio of CD14^++^CD80^+^ cells/CD14^++^CD163^+^ cells were elevated, implying the localization of inflammation in the IFP. Therefore, targeting adipose-tissue-resident innate immune cells in the IFP can be considered as a new therapeutic strategy for inflammatory arthritis.

## Data Availability Statement

The original contributions presented in the study are included in the article/**Supplementary Material**. Further inquiries can be directed to the corresponding author.

## Ethics Statement

The studies involving human participants were reviewed and approved by the Kyoto University Graduate School and Faculty of Medicine Ethics Committee (approval number G0502-1). The patients/participants provided their written informed consent to participate in this study.

## Author Contributions

SM and KosM were responsible for the study design. SM, RS, and MTani collected samples during total knee arthroplasty. SM and RS carried out the experiments. HI, KoiM, and KN performed the surgery. SM wrote the first draft of the manuscript. MH, MTana, KK, SA, RN, HY, KO, AM, and TM supervised the draft of the manuscript. All authors contributed to the article and approved the submitted version.

## Funding

This work was supported by Nagahama City, Shiga, Japan; Toyooka City, Hyogo, Japan; and five pharmaceutical companies (Mitsubishi Tanabe Pharma Co., Chugai Pharmaceutical Co. Ltd., UCB Japan Co. Ltd., AYUMI Pharmaceutical Co., and Asahi Kasei Pharma Co.). The KURAMA cohort study is supported by grants from Daiichi Sankyo Co. Ltd. This study was conducted as an investigator-initiated study. The companies had no role in the design of the study, the collection or analysis of the data, the writing of the manuscript, or the decision to submit the manuscript for publication.

## Conflict of Interest

The authors declare that the research was conducted in the absence of any commercial or financial relationships that could be construed as a potential conflict of interest.

## Publisher’s Note

All claims expressed in this article are solely those of the authors and do not necessarily represent those of their affiliated organizations, or those of the publisher, the editors and the reviewers. Any product that may be evaluated in this article, or claim that may be made by its manufacturer, is not guaranteed or endorsed by the publisher.
