# Supplementary material for: Increased Ratio of CD14++CD80+ Cells/CD14++CD163+ Cells in the Infrapatellar Fat Pad of End-Stage Arthropathy Patients
Source: Front Immunol. 2021 Nov 26;12:774177. doi: 10.3389/fimmu.2021.774177 (PMC8662627; doi:10.3389/fimmu.2021.774177)
Supplement: Supplementary file 5 [file Table_1.docx]

***Supplementary Material***

**Supplementary Tables**

|  | **OA (n=15)** | **RA_Others (n=7)** | **RA_bDMARDs (n=9)** |
| --- | --- | --- | --- |
| **IFP** | 4.15 x 10^5^ (3.87 x 10^5^) | 2.73 x 10^5^ (1.80 x 10^5^) | 3.44 x 10^5^ (3.63 x 10^5^) |
| **SC** | 1.78 x 10^4^ (1.91 x 10^4^) | 1.46 x 10^4^ (9.73 x 10^3^) | 4.67 x 10^4^ (5.14 x 10^4^) |

**Supplementary Table 1.** **The amounts of cells extracted RNA for qPCR from OA and RA patients.** Mean (SD) are displayed. IFP: infrapatellar fat pad; SC: subcutaneous fat tissue

| **Gene** | **Forward Sequences (5'-3')** | **Reverse Sequences (5'-3')** | **Reference** |
| --- | --- | --- | --- |
| **Human** | | |  |
| *GAPDH* | TGCACCACCAACTGCTTAGC | GGCATGGACTGTGGTCATGAG | (22) |
| *SREBP1A* | ATCGAAGGTGAAGTCGGCG | CCAGCATAGGGTGGGTCAAA | This paper |
| *SREBP1C* | ATGCGGAGGGTGTTCCTACAT | CAGACTGCGGTCGAGGAGC | This paper |
| *IL6* | CATCCTCGACGGCATCTCAG | CCAGGCAAGTCTCCTCATTGAA | This paper |
| *IL1B* | CTCGCCAGTGAAATGATGGCT | GTCGGAGATTCGTAGCTGGAT | (23) |
| *CXCL10* | GTGGCATTCAAGGAGTACCTC | GCCTTCGATTCTGGATTCAGACA | (24) |
| *NR1H3* (*LXRA*) | GTTATAACCGGGAAGACTTTGCCA | GCCTCTCTACCTGGAGCTGGT | (25) |

**Supplementary Table 2.** Primers sequences for running quantitative real-time polymerase chain-reaction.

|  |  | **CD14 positive cells (10^5^)/grams of fat tissue** | **M1/M2 ratio** | **SREBP1A** | **SREBP1C** | **LXRA** | **CXCL10** | **IL1B** | **IL6** |
| --- | --- | --- | --- | --- | --- | --- | --- | --- | --- |
| **OA** | Age<77 | **0.0078** | 0.1143 | 0.3125 | **0.0313** | **0.0313** | 0.0938 | 0.5625 | 0.5625 |
|  |  | N=9 | N=4 | N=6 | | | | | |
|  | Age≥77 | **9.77E-04** | Not assessed | 0.5703 | 0.4961 | 0.0742 | 0.1953 | 0.7344 | 0.0742 |
|  |  | N=11 | N=0 | N=9 | | | | | |
| **RA without bDMARDs** | Age<68 | 0.2188 | 0.8000 | 0.2500 | 0.2500 | 0.2500 | 0.7500 | 1.000 | 0.5000 |
|  |  | N=7 | N=2 | N=3 | | | | | |
|  | Age≥68 | **0.0156** | Not assessed | 0.2500 | 0.2500 | 0.1250 | 0.8750 | 0.3750 | 0.8750 |
|  |  | N=6 | N=1 | N=4 | | | | | |
| **RA with bDMARDs** | Age<68 | 0.8125 | Not assessed | 0.8750 | 0.2500 | 0.1250 | 0.6250 | 0.3750 | 0.3750 |
|  |  | N=5 | N=1 | N=4 | | | | | |
|  | Age≥68 | 0.0625 | Not assessed | 0.2500 | 0.8750 | 0.8750 | 0.1250 | 0.6250 | 0.6250 |
|  |  | N=5 | N=1 | N=4 | | | | | |

**Supplementary Table 3.** Impact of age category in OA and RA patients on the related gene expression. Patients were classified into age groups below or above the median age in each disease. Statistics of CD14 positive cells and gene expression levels in each group were determined by paired sample Wilcoxon signed rank, while statistics for the M1/M2 ratio in each group were determined using Mann-Whitney U tests. Data are expressed as p-values between SC and IFP. Data in bold font indicate significance.
